# Long-Term Peri-Implant Outcomes of Smoother/Hybrid Versus Moderately Rough Titanium Implant Surfaces: An Updated Systematic Review and Meta-Analysis

**DOI:** 10.7759/cureus.115357

**Published:** 2026-08-28

**Authors:** Heliette Emperatriz E Delgado Castromonte, Arturo P Jaramillo, Juan S Hernandez Jiménez, Kareen Mercado Vega, Misael Martinez Becerra, Wellinger Vera, Angela Alcala

**Affiliations:** 1 Dentistry, Universidad Gran Mariscal de Ayacucho, Anzoategui, VEN; 2 General Practice, Universidad Estatal de Guayaquil, Machala, ECU; 3 Dentistry, Universidade Federal de Minas Gerais, Belo Horizonte, BRA; 4 Business Health Management, Pontifícia Universidade Católica do Paraná, Curitiba, BRA; 5 Dentistry, Universidad Nacional de Colombia, Bogota, COL; 6 Periodontics, Institución Universitaria Colegios de Colombia, Bogota, COL; 7 General Dentistry, Universidad de Ciencias Médicas de La Habana “Victoria de Girón”, La Habana, CUB; 8 Dentistry, Universidad José Antonio Páez, Carabobo, VEN; 9 Dentistry, Paulo Picanço School of Dentistry, Ceará, BRA

**Keywords:** dental implants paper reviewer, implant surface, marginal bone loss, peri-implantitis, surface roughness and microhardness

## Abstract

Roughened titanium implant surfaces can improve osseointegration, whereas smoother or hybrid coronal surfaces may limit biofilm retention if exposed. Whether either strategy improves long-term peri-implant outcomes remains uncertain. This update compared smoother, minimally rough, or hybrid implant surfaces with moderately rough or rougher surfaces after at least 24 months of follow-up. A 2024 systematic review was used as the baseline evidence set. PubMed was updated from January 1, 2024, through August 7, 2026, with forward and backward citation searching. Randomized clinical reports in adults were eligible when the surface contrast was not inseparable from a different whole implant system or collar macrodesign. The primary outcome was marginal bone-level change (MBL); probing depth (PPD) and implant failure were secondary outcomes. Risk of bias was assessed with RoB 2 (The Cochrane Collaboration, London, UK). RevMan 5.4 (The Cochrane Collaboration, London, UK)-compatible inverse-variance random-effects models were used for continuous outcomes, and a Mantel-Haenszel fixed-effect risk ratio was used for sparse implant-failure events. Ten reports involving 480 enrolled participants and 1,219 implants met the review criteria. Eight reports contributed to the MBL meta-analysis (671 analysis units). There was no evidence of a difference between smoother/hybrid and rougher surfaces (mean difference (MD) 0.07 mm, 95% confidence interval (CI) -0.12 to 0.27; p=0.46; I²=84%). Four reports contributed PPD data (MD -0.36 mm, 95% CI -0.93 to 0.21; p=0.22; I²=72%). Implant failure was uncommon (12/381 versus 6/392 implants); the risk ratio was imprecise and not statistically significant (RR 2.12, 95% CI 0.85 to 5.31; p=0.11; I²=0%). Excluding studies at high risk of bias did not materially alter the MBL conclusion. Current randomized evidence does not establish long-term clinical superiority of smoother/hybrid or rougher titanium implant surfaces. Confidence is very low because of risk of bias, substantial clinical and statistical heterogeneity, sparse failures, and unresolved clustering in multi-implant and split-mouth trials. Surface selection should therefore be individualized and should not rely on roughness alone.

## Introduction and background

Dental implants are a predictable option for replacing missing teeth, but biological complications can occur after loading. Peri-implantitis is defined as a plaque-associated pathological condition characterized by inflammation in the peri-implant mucosa and progressive loss of supporting bone [1]. Preservation of marginal bone and peri-implant soft-tissue health is therefore central to long-term treatment success.

Implant surface topography influences protein adsorption, cellular attachment, bone-to-implant contact, and microbial retention. Surface roughness is commonly summarized by the arithmetical mean height (Sa), although chemistry, wettability, and the spatial location of the modified surface also contribute to biological behavior [2]. Moderately rough surfaces may support early osseointegration, particularly in compromised bone or accelerated loading protocols. Conversely, when the coronal implant surface becomes exposed, a rougher microtopography may be more difficult to decontaminate and may favor mature biofilm retention. Hybrid designs attempt to reconcile these competing considerations by combining a smoother coronal region with a rougher implant body.

Earlier reviews have produced inconsistent conclusions. A long-term meta-analysis found only small average differences in crestal bone loss across roughness categories and emphasized confounding by implant geometry, connection design, loading, and patient factors [3]. A 2024 review of 10 randomized trials reported no significant difference in marginal bone loss, a possible reduction in probing depth with machined surfaces, and a non-significant tendency toward fewer failures with rougher surfaces [4]. However, that synthesis combined several comparisons in which surface roughness was inseparable from broader implant-system or collar-design differences. It also preceded new long-term randomized reports published in 2024 and 2025.

The present work was designed as a focused update of that evidence base. The objective was to compare long-term marginal bone-level change, peri-implant probing depth, and implant failure between smoother/minimally rough or hybrid titanium implant surfaces and moderately rough or rougher surfaces, while excluding whole-system comparisons in which the surface effect could not be reasonably separated from macrodesign. The null hypothesis was that the two surface strategies would not differ for any prespecified outcome.

## Review

Materials and methods

Protocol and Reporting Standard

This systematic review update was prepared in accordance with the Preferred Reporting Items for Systematic Reviews and Meta-Analyses (PRISMA) 2020 statement [5]. It was not prospectively registered. Because the work was an update rather than a de novo review, the eligible-study set from Hussein et al. was used as the baseline and was reappraised under the narrower criteria described below [4].

Focused Question and Eligibility Criteria

The PICOS question was: in adults receiving endosseous titanium dental implants (population), do machined, turned, minimally rough, or hybrid implants with a smoother coronal component (intervention), compared with moderately rough or rougher implants (comparison), produce different long-term MBL, PPD, peri-implant disease, or implant-failure outcomes (outcomes) in randomized clinical trials (study design)?

The prespecified eligibility criteria applied during study selection are summarized in Table 1. For multiple publications arising from the same trial cohort, only the report with the longest eligible follow-up was retained.

**Table 1:** Eligibility criteria for study selection. MBL, marginal bone-level change; PPD, peri-implant probing depth.

Eligibility domain	Inclusion criterion	Exclusion criterion
Population	Adults receiving root-form titanium dental implants.	Animal, in vitro, or non-adult studies.
Study design	Randomized clinical trials comparing implant-surface strategies.	Observational, non-randomized, case-series, or review designs.
Surface comparison	Smoother, machined, turned, minimally rough, or hybrid surfaces compared with moderately rough or rougher surfaces.	Studies without a direct eligible surface comparison.
Implant comparability	Arms with sufficiently similar implant geometry and prosthetic connection to permit interpretation of the surface contrast.	Comparisons in which different whole implant systems or collar macrodesigns made the surface effect inseparable from other design features.
Follow-up	At least 24 months of clinical follow-up after implant placement or loading.	Follow-up shorter than 24 months.
Outcomes and data	Group-level numerical data for MBL, PPD, peri-implant disease, or implant failure.	No extractable group-level quantitative outcome data.
Publication availability	English-language full text.	Non-English reports or reports without an accessible full text.
Multiple reports from one cohort	The longest eligible follow-up report from each trial cohort.	Earlier eligible reports superseded by a longer follow-up from the same cohort.

Information Sources and Search Strategy

The baseline evidence set was obtained from Hussein et al., whose systematic review searched PubMed, Embase, and Scopus through January 2024 [4]. For the present update, PubMed was searched from January 1, 2024, through August 7, 2026, and forward and backward citation searches were conducted for the baseline review and eligible long-term trials. The information sources, date limits, search procedures, and yields are presented in Table 2.

**Table 2:** Information sources and search strategies used for the updated review. The exact database-specific strategies for the baseline review were not rerun and are reported in Hussein et al. [4].

Source	Coverage	Search strategy or procedure	Yield
Baseline systematic review [4]	Through January 2024	The eligible-study set from a review covering PubMed, Embase, and Scopus was reappraised under the narrower criteria of the present update.	10 reports
PubMed update	January 1, 2024-August 7, 2026	(dental implant*[Title/Abstract]) AND (surface roughness[Title/Abstract] OR rough surface*[Title/Abstract] OR machined surface*[Title/Abstract] OR hybrid surface*[Title/Abstract] OR implant surface*[Title/Abstract]) AND (randomized controlled trial[Publication Type] OR random*[Title/Abstract])	72 records
Forward and backward citation searching	Through August 7, 2026	Reference lists and citing records for the baseline review and eligible long-term trials were screened for additional randomized reports.	2 records

Across the update methods, 74 records were identified (72 in PubMed and two through citation searching), and no duplicate records were removed. After title and abstract screening, five reports underwent full-text assessment; two were excluded because follow-up was shorter than 24 months. The 10 reports from the baseline review were also reappraised under the current eligibility criteria. Three reports (Astrand 2004, Moberg 2001, and Camarda 2021) were excluded because the surface treatment could not be separated from broader implant-system or collar-macrodesign differences. Seven earlier reports were retained, and three newly eligible reports were added, yielding 10 reports in the updated review.

Study Selection and Data Extraction

One reviewer conducted update screening, full-text eligibility assessment, and data extraction. Decisions were cross-checked against the earlier review's study table and the primary full texts. No independent duplicate screening or extraction was performed; this departure from preferred review practice was prespecified as a limitation and informed the certainty assessment.

Extracted variables were publication year, design, number of randomized and analyzed participants, number of implants, surface characteristics, follow-up, analysis unit, MBL, PPD, and implant failures. MBL was standardized so that a higher positive value represented greater bone loss. For continuous outcomes, the effect was defined as smoother/hybrid minus rougher; negative values therefore favored smoother/hybrid surfaces. For implant failure, a risk ratio (RR) below 1 favored smoother/hybrid surfaces. The longest eligible time point was extracted.

Risk of Bias and Certainty of Evidence

Risk of bias was assessed at the outcome level with the Cochrane RoB 2 (The Cochrane Collaboration, London, UK) framework across randomization, deviations from intended interventions, missing data, outcome measurement, and selective reporting [6]. Overall judgments were low risk, some concerns, or high risk.

Statistical Analysis

Calculations reproduced the published Review Manager 5 statistical algorithms (The Cochrane Collaboration, London, UK) [7]. Mean differences and 95% CIs were pooled with inverse-variance DerSimonian-Laird random-effects models because clinical heterogeneity was expected. Implant failure was pooled with a Mantel-Haenszel fixed-effect RR because events were sparse and statistical heterogeneity was absent. A continuity correction of 0.5 was applied to all four cells in single-zero studies; double-zero studies were displayed but were not estimable for the RR. Statistical heterogeneity was assessed with Cochran's Q and I². Two-sided p<0.05 denoted statistical significance.

One split-mouth report provided a paired PPD estimate and its 95% CI; this estimate was analyzed using generic inverse variance. Several split-mouth and multi-implant reports did not provide the paired standard deviation or an intracluster correlation. Their arm-level MBL data were therefore analyzed as reported, without a design-effect adjustment. This may overstate precision and is explicitly addressed in the limitations. A sensitivity analysis excluded reports judged at high risk of bias. Because fewer than 10 reports contributed to each outcome, no formal test of funnel-plot asymmetry was performed. An exploratory funnel plot was generated for MBL to visualize the distribution of effect estimates against their standard errors; it was interpreted descriptively and was not used to infer publication bias.

Results

Study Selection

**Figure 1:**
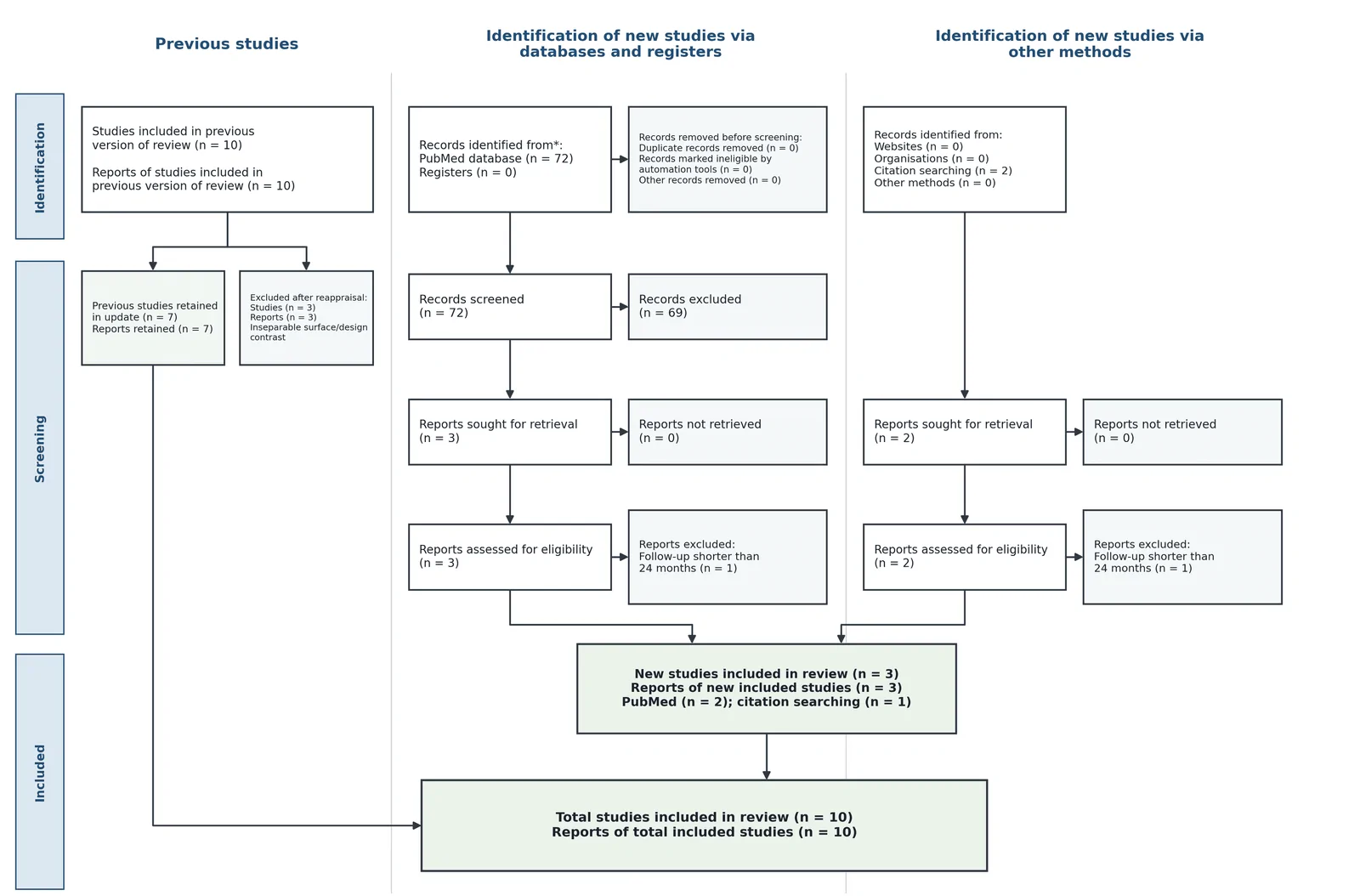
PRISMA Diagram Preferred Reporting Items for Systematic Reviews and Meta-Analyses (PRISMA) 2020 flow diagram for the updated systematic review. No study registers were searched; all database records were identified in PubMed. The database and citation-search pathways are reported separately in accordance with the official updated-review template. Adapted from the PRISMA 2020 statement [5].

The study-selection process is summarized in Figure 1. Of the 10 studies represented by 10 reports in the previous review, three were excluded after eligibility reappraisal because the implant-surface contrast was inseparable from differences in implant system or collar macrodesign, leaving seven studies for inclusion in the update. The updated PubMed search identified 72 records; 69 were excluded during title and abstract screening, and three full-text reports were assessed for eligibility. One report was excluded because the follow-up period was shorter than 24 months, leaving two newly eligible studies. Citation searching identified two additional reports, both of which were retrieved and assessed; one was excluded for having less than 24 months of follow-up, and one met the eligibility criteria. Consequently, three new studies were added to the seven retained studies, resulting in a final evidence set of 10 studies represented by 10 reports (Figure 1).

Study Characteristics

The designs, populations, surface comparisons, follow-up periods, and quantitative contributions of the 10 included randomized clinical reports are summarized in Table 3.

**Table 3:** Characteristics of the 10 included randomized clinical reports. MBL, marginal bone-level change; PPD, probing pocket depth; RCT, randomized controlled trial.

Study	Design/population	Enrolled participants / implants	Surface comparison	Longest follow-up	Quantitative contribution
Donati et al., 2018 [8]	Randomized clinical trial; history of periodontitis represented	25 / 64	Turned/minimally rough vs TiOblast/moderately rough	240 months	MBL, PPD
Zetterqvist et al., 2010 [9]	Prospective multicenter RCT	112 / 304	Hybrid machined coronal third vs continuously etched	60 months	Peri-implant outcomes; narrative
Rocci et al., 2013 [10]	Randomized open-ended trial; posterior mandible	44 / 121	Machined vs anodically oxidized TiUnite	108 months	MBL, failure
Tirone et al., 2021 [11]	Double-blind split-mouth RCT	114 / 228	Machined vs oxidized/roughened	36 months	MBL, failure
Rothamel et al., 2022 [12]	Randomized multicenter trial	58 / 116	Machined vs structured implant shoulder	24 months	MBL, failure
Nicu et al., 2012 [13]	Pilot RCT	14 / 78	Turned vs anodically oxidized	36 months	PPD
Raes et al., 2018 [14]	Split-mouth RCT; severe periodontitis	15 / 84	Minimally rough vs moderately rough	60 months	MBL, PPD, failure
Glibert et al., 2024 [15]	Split-mouth RCT; mandibular overdenture	24 / 48 (21 / 42 analyzed)	Hybrid 3-mm machined coronal zone vs fully moderately rough	Mean 73 months	MBL, paired PPD, failure
Serrano et al., 2025 [16]	Parallel RCT; treated periodontitis	40 / 40 (36 analyzed)	Hybrid machined collar vs moderately rough to shoulder	36 months	MBL, failure
Offord et al., 2025 [17]	Split-mouth RCT; immediately loaded maxillary full arch	34 / 136 (24 / 94 analyzed)	Hybrid 3-mm minimally rough coronal zone vs fully moderately rough	Mean 53 months	MBL, failure

Across the 10 reports, 480 participants and 1,219 implants were enrolled. Follow-up ranged from 24 months to 20 years. Populations varied from periodontally healthy fully edentulous patients to participants with treated severe periodontitis. Prosthetic protocols included single crowns, partial fixed restorations, mandibular overdentures, and immediately loaded maxillary full-arch prostheses. Surface contrasts also varied: some trials compared turned with oxidized surfaces over the implant body, whereas newer trials compared a hybrid machined coronal segment with a fully moderately rough implant. This clinical diversity anticipated heterogeneity.

Risk of Bias

Outcome-level RoB 2 judgments for the 10 included reports are summarized in Table 4.

**Table 4:** RoB 2 judgments for the 10 included reports. Low, low risk of bias; Some concerns, some concerns; High, high risk of bias. RoB 2, risk of bias 2 tool by the Cochrane Collective, London, UK.

Study	Randomization	Deviations	Missing data	Measurement	Selective reporting	Overall
Donati et al., 2018 [8]	Low	Low	Low	Low	Low	Low
Zetterqvist et al., 2010 [9]	Some concerns	Low	Some concerns	Low	Some concerns	Some concerns
Rocci et al., 2013 [10]	Some concerns	Some concerns	High	Some concerns	Some concerns	High
Tirone et al., 2021 [11]	Low	Low	Some concerns	Low	Some concerns	Some concerns
Rothamel et al., 2022 [12]	Some concerns	Low	High	Low	Some concerns	High
Nicu et al., 2012 [13]	Some concerns	Low	High	Low	Some concerns	High
Raes et al., 2018 [14]	Low	Low	Some concerns	Low	Some concerns	Some concerns
Glibert et al., 2024 [15]	Some concerns	Low	Some concerns	Low	Some concerns	Some concerns
Serrano et al., 2025 [16]	Low	Low	Low	Low	Low	Low
Offord et al., 2025 [17]	Low	Low	High	Low	Some concerns	High

Two reports were judged at low risk of bias, four raised some concerns, and four were judged at high risk. The most important limitations were incomplete reporting of allocation methods in older trials, attrition at long-term follow-up, and incomplete availability of prespecified analysis plans. Blinded radiographic outcome measurement reduced concern in several newer reports. Commercial support or provision of implant components was common; this was not itself treated as a RoB 2 domain but was considered when interpreting applicability.

Marginal Bone-Level Change

**Figure 2:**
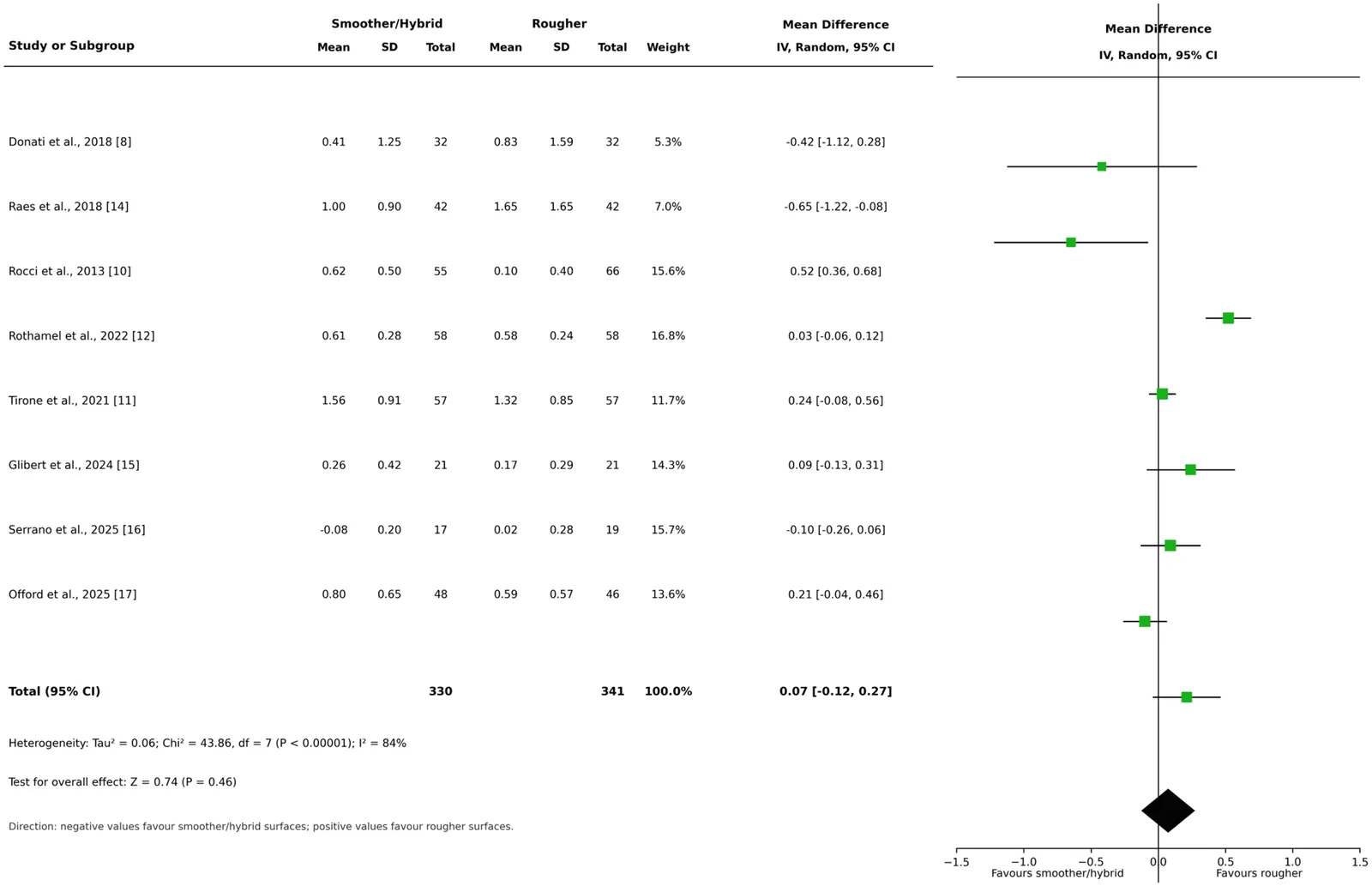
Forest plot of marginal bone-level change. Mean differences were calculated as smoother/hybrid minus rougher. Negative values favor smoother/hybrid surfaces, whereas positive values favor rougher surfaces. Each study label includes its corresponding reference number. CI, confidence interval; IV, inverse variance; MD, mean difference.

Eight reports contributed MBL data for 330 smoother/hybrid and 341 rougher analysis units. The pooled mean difference (MD) was 0.07 mm (95% CI -0.12 to 0.27; Z=0.74; p=0.46), indicating no statistically significant difference (Figure 2). Heterogeneity was substantial (Tau²=0.06; Q=43.86, df=7, p<0.00001; I²=84%). The point estimate was small relative to typical radiographic measurement error and did not support clinically meaningful superiority of either surface strategy.

When the three MBL reports at high risk of bias were excluded, the pooled estimate remained non-significant (MD -0.06 mm, 95% CI -0.29 to 0.16; p=0.59), and heterogeneity decreased but remained important (I²=61%). Thus, the primary conclusion was not driven by the high-risk reports.

Peri-Implant Probing Depth

**Figure 3:**
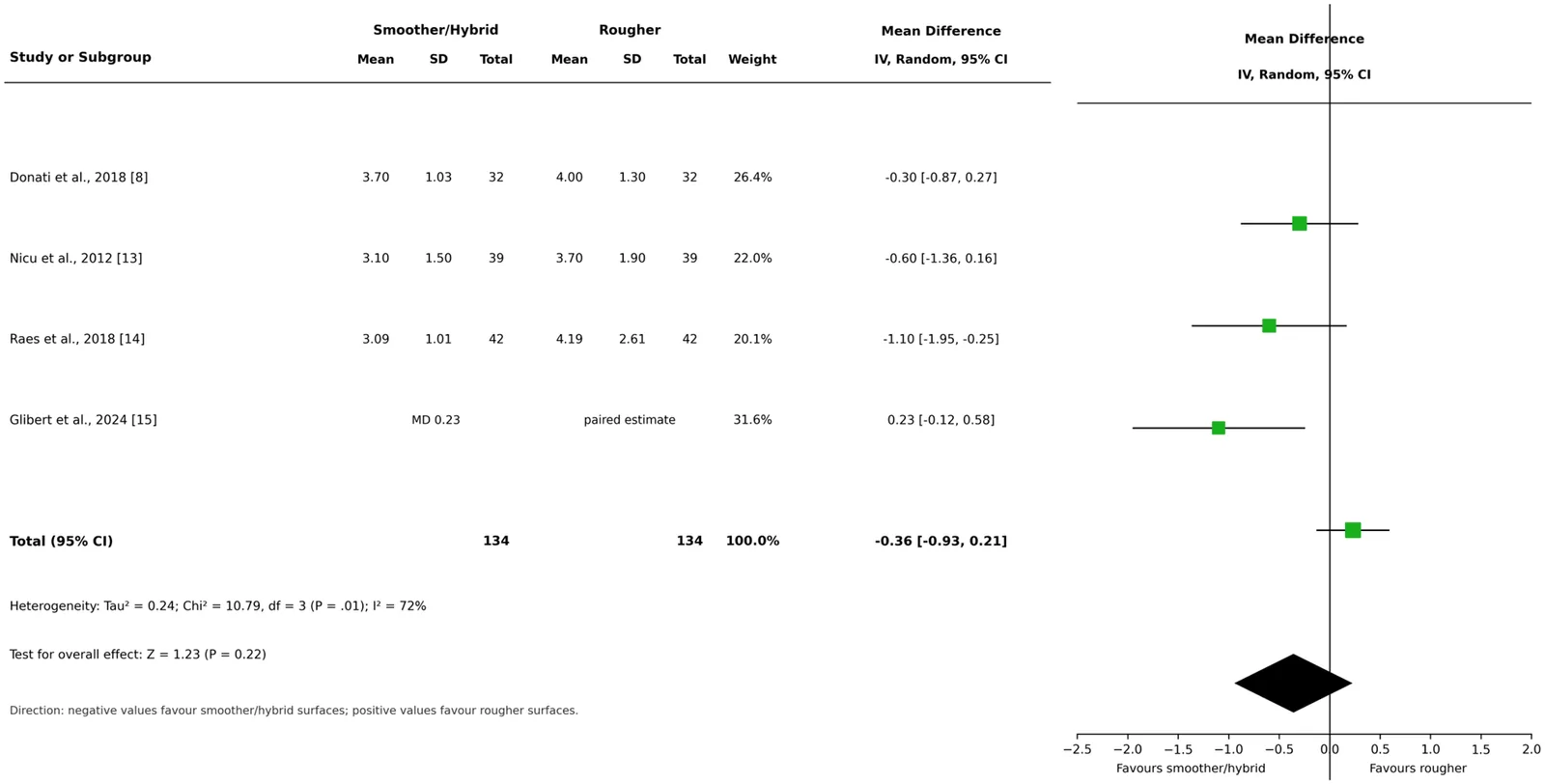
Forest plot of peri-implant probing depth. Negative MD values favor smoother/hybrid surfaces, whereas positive values favor rougher surfaces. The Glibert estimate was entered using generic inverse variance from the reported paired MD and 95% CI. Each study label includes its corresponding reference number. CI, confidence interval; IV, inverse variance; MD, mean difference.

Four reports contributed PPD information for 134 analysis units per surface strategy. For Glibert et al. [15], the paired PPD estimate and its 95% CI entered using generic inverse variance The random-effects MD was -0.36 mm (95% CI -0.93 to 0.21; Z=1.23; p=0.22), with substantial heterogeneity (Tau²=0.24; Q=10.79, df=3, p=0.01; I²=72%) (Figure 3). The CI included both a potentially relevant reduction in PPD with smoother/hybrid surfaces and no difference.

Implant Failure

**Figure 4:**
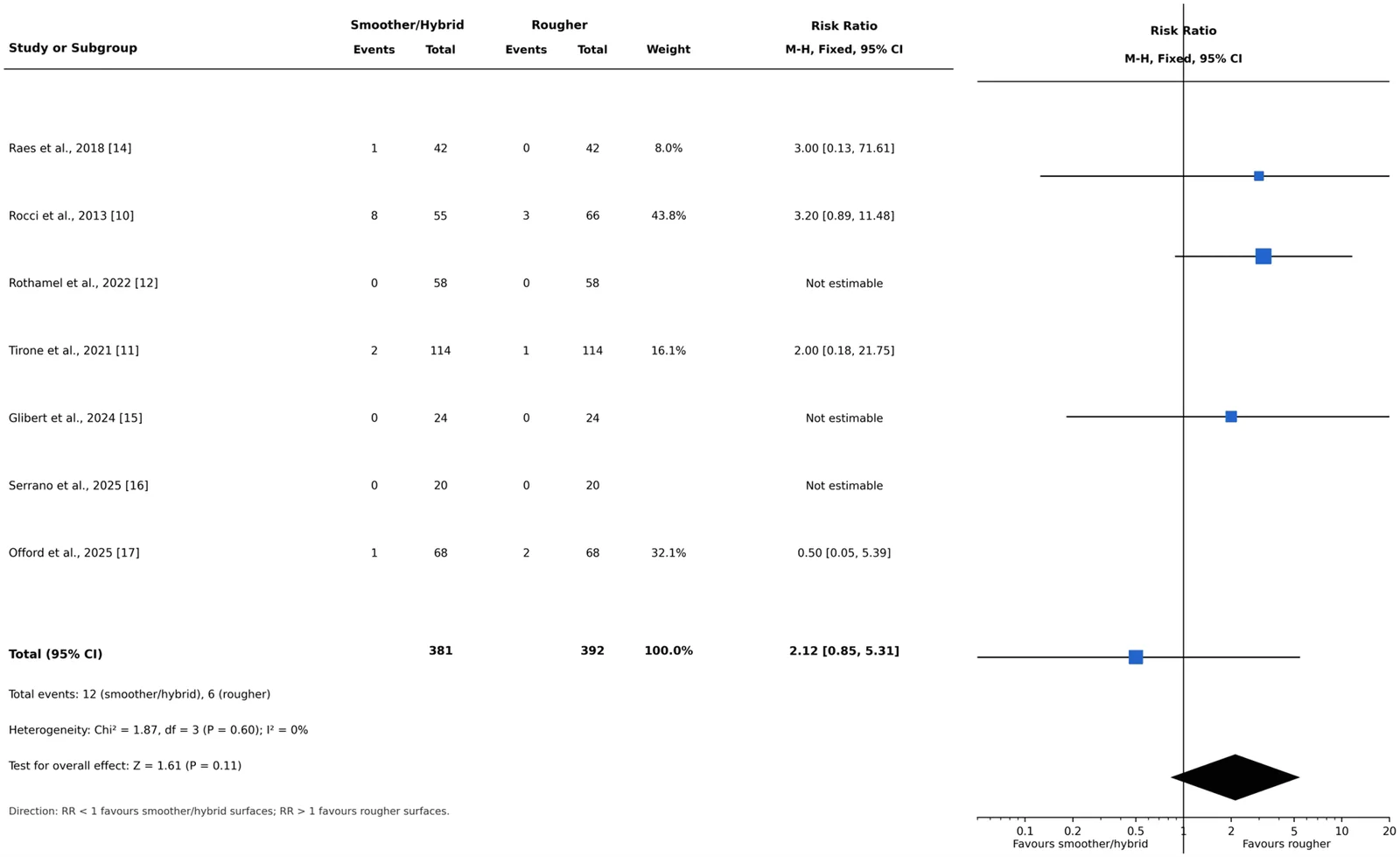
Forest plot of implant failure. RR values below 1 favor smoother/hybrid surfaces, whereas RR values above 1 favor rougher surfaces. Double-zero studies are displayed but are not estimable. Each study label includes its corresponding reference number. CI, confidence interval; M-H, Mantel-Haenszel; RR, risk ratio.

Seven reports provided implant-failure counts for 381 smoother/hybrid and 392 rougher implants. Three reports had zero failures in both arms and were not estimable for the RR. Overall, 12 failures occurred in the smoother/hybrid groups and six in the rougher groups. The Mantel-Haenszel RR was 2.12 (95% CI 0.85 to 5.31; Z=1.61; p=0.11), with no detected heterogeneity (Q=1.87, df=3, p=0.60; I²=0%) (Figure 4). The wide CI and low event count precluded a reliable conclusion about relative failure risk.

Funnel Plot Diagram

**Figure 5:**
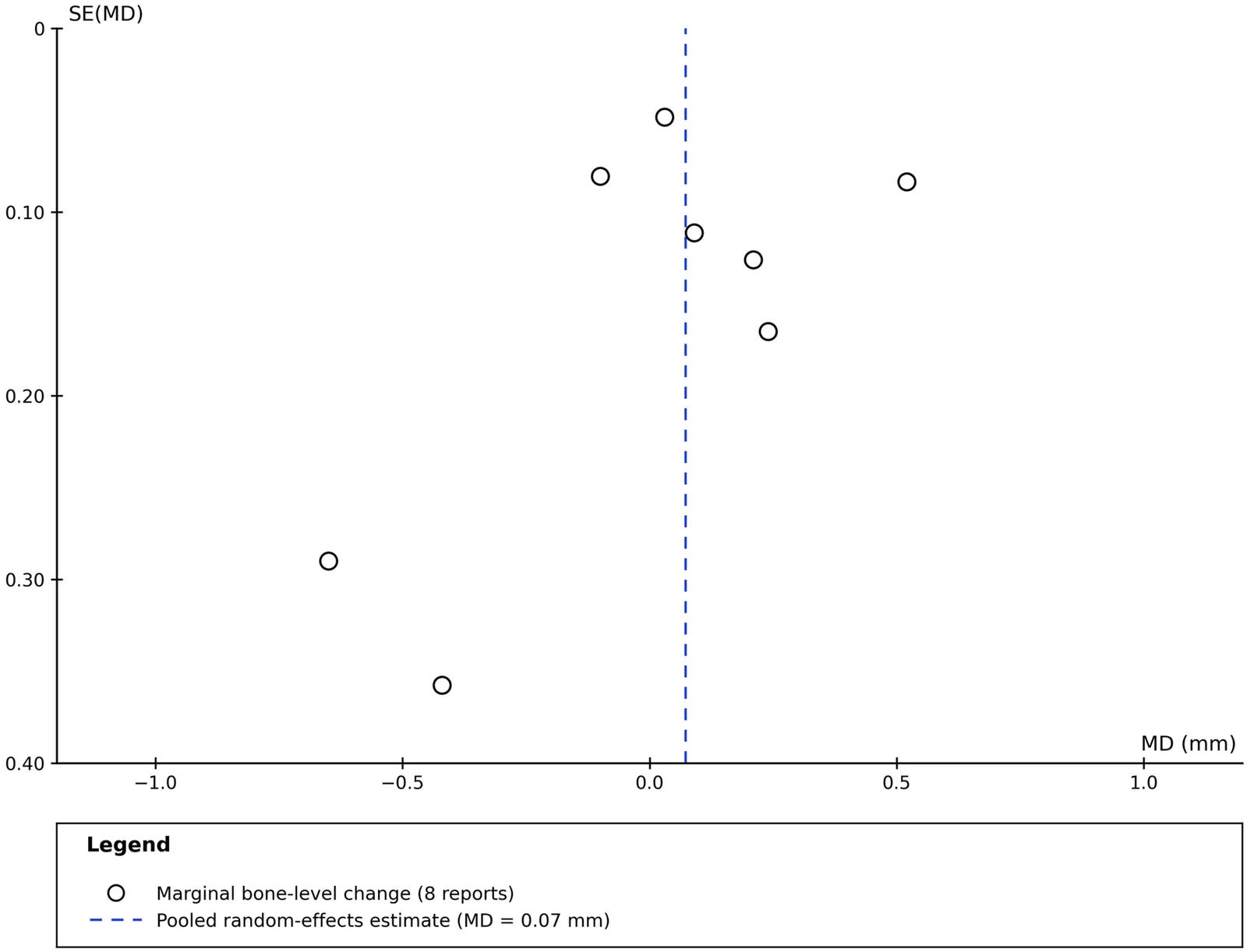
Funnel plot of marginal bone-level change. Each open circle represents an individual report, and the dashed blue line indicates the pooled random-effects MD of 0.07 mm. Negative values favor smoother/hybrid surfaces, whereas positive values favor rougher surfaces. MD, mean difference; SE, standard error.

An exploratory funnel plot was generated for the primary MBL outcome (Figure 5). Visual inspection suggested modest asymmetry, mainly because two less precise reports had negative effect estimates and one comparatively precise report had a positive estimate. However, only eight reports contributed to this analysis, and substantial between-study heterogeneity was present (I²=84%). The distribution may therefore reflect differences in implant characteristics, radiographic assessment methods, and follow-up periods rather than selective publication. The plot should be interpreted descriptively and does not provide sufficient evidence to establish publication bias.

Summary of Findings

The pooled effects, heterogeneity estimates, and certainty judgments for the three prespecified outcomes are summarized in Table 5.

**Table 5:** Summary of quantitative findings and certainty. CI, confidence interval; IV, inverse variance; M-H, Mantel-Haenszel; MD, mean difference; RR, risk ratio.

Outcome	Reports / analysis units	Model	Pooled effect (95% CI)	Heterogeneity	Certainty / interpretation
Marginal bone-level change	8 / 671	IV random effects	MD 0.07 mm (-0.12 to 0.27)	I²=84%	Very low; no demonstrated difference
Probing depth	4 / 268	IV random effects	MD -0.36 mm (-0.93 to 0.21)	I²=72%	Very low; effect uncertain
Implant failure	7 / 773	M-H fixed effect	RR 2.12 (0.85 to 5.31)	I²=0%	Very low; sparse events and wide CI

Discussion

Principal Findings

This update found no convincing long-term advantage of smoother/hybrid or rougher titanium implant surfaces for MBL, PPD, or implant failure. The pooled MBL difference was only 0.07 mm, and its CI crossed the null. PPD was nominally lower with smoother/hybrid surfaces, but the estimate was imprecise and heterogeneous. Implant failures were numerically more frequent with smoother/hybrid surfaces; however, only 18 failures occurred, and the CI ranged from a possible reduction to more than a five-fold increase. The evidence therefore supports equipoise rather than a claim of clinical equivalence or superiority.

The MBL finding is broadly consistent with the 2024 synthesis, which also found no significant difference [4]. The present PPD result is more conservative because the eligibility reappraisal excluded confounded collar/system comparisons and incorporated a recent paired long-term estimate. The high I² values indicate that a single average effect should not be generalized across all implant systems, patient risk profiles, and loading protocols.

Biological and Clinical Interpretation

Surface roughness presents a biological tradeoff. A rougher surface increases available topographic area and can improve early mechanical interlocking and bone apposition. Those properties may be valuable when primary stability, bone density, or loading time is challenging. Conversely, exposure of a rough coronal surface after remodeling or disease may increase microbial retention and complicate decontamination. Hybrid implants seek to preserve a rough osseointegrating body while limiting coronal roughness. The current results suggest that this rationale does not translate into a reproducible long-term benefit across the available randomized trials.

The absence of a large average difference does not mean that surface is irrelevant in every patient. In a review restricted to patients with a history of periodontal disease, differences in survival and MBL were also uncertain, and the authors emphasized limited long-term data and uncontrolled confounding [18]. Another review found that peri-implantitis prevalence estimates differed across turned and rough surfaces, but heterogeneity in disease definitions and maintenance protocols prevented firm causal inference [19]. Patient-level determinants - history of periodontitis, smoking, plaque control, maintenance adherence, restorative contour, and residual cement - may exert greater influence than the surface category alone.

Clinically, implant selection should therefore integrate bone quality, loading strategy, implant macrogeometry, connection design, restorative space, periodontal risk, and the feasibility of lifelong supportive care. The pooled failure estimate should not be used to avoid hybrid implants or to mandate rougher implants; the number of events is too small for that conclusion. Similarly, the non-significant PPD estimate is insufficient to prefer a smoother coronal surface for disease prevention.

Strengths

This update used a prespecified minimum follow-up, retained only the longest report from each cohort, and reappraised whether the surface contrast could be interpreted independently of whole-system or collar macrodesign. The review incorporated three newer long-term reports and maintained an auditable final set of exactly 10 randomized reports. Effect directions were harmonized, sparse events were analyzed with a Mantel-Haenszel method, double-zero studies were displayed transparently, and a sensitivity analysis tested the influence of high-risk reports. Formal funnel-plot asymmetry testing was appropriately withheld because fewer than 10 reports contributed to each outcome, while the exploratory MBL funnel plot was interpreted descriptively.

Limitations

Overall certainty was summarized descriptively using the Grading of Recommendations Assessment, Development, and Evaluation (GRADE) domains of risk of bias, inconsistency, indirectness, imprecision, and publication bias [20].

This review has two principal limitations. First, the updated search was limited to PubMed and citation tracking and was conducted by one reviewer, although the previous systematic review provided Embase and Scopus coverage through January 2024. Therefore, the possibility that some eligible reports were missed cannot be completely excluded. Second, differences in radiographic assessment methods, follow-up periods, implant-surface characteristics, and the handling of multiple implants within individual participants contributed to heterogeneity and should be considered when interpreting the pooled estimates. Nevertheless, the primary conclusion remained stable after sensitivity analysis, supporting the overall consistency and clinical relevance of the findings.

Research Implications

Future trials should vary surface topography while holding macrogeometry, implant-abutment connection, placement depth, and prosthetic protocol constant. They should prespecify a contemporary peri-implantitis definition, report patient- and implant-level outcomes, provide paired or cluster-adjusted estimates, and retain participants for at least five to 10 years. Core outcomes should include standardized MBL change from loading, PPD, bleeding/suppuration, peri-implantitis, implant survival, maintenance burden, and patient-reported outcomes. Individual-participant-data meta-analysis would be particularly valuable for exploring periodontitis history, smoking, maintenance adherence, and loading as effect modifiers.

## Conclusions

Among 10 randomized clinical reports with at least 24 months of follow-up, smoother/minimally rough or hybrid titanium implant surfaces did not produce a statistically significant difference in marginal bone-level change or probing depth compared with moderately rough or rougher surfaces. Implant failure was uncommon, and the apparent numerical advantage of rougher surfaces was too imprecise for a firm conclusion. The certainty of evidence is very low because of risk of bias, substantial heterogeneity, sparse events, and unresolved clustering. Surface roughness should be considered as one component of implant selection-not as an isolated determinant of long-term peri-implant health.
